# Insights into Orphan Nuclear Receptors as Prognostic Markers and Novel Therapeutic Targets for Breast Cancer

**DOI:** 10.3389/fendo.2015.00115

**Published:** 2015-08-07

**Authors:** Reidun Aesoy, Colin D. Clyne, Ashwini L. Chand

**Affiliations:** ^1^Cancer Drug Discovery, Hudson Institute of Medical Research, Melbourne, VIC, Australia; ^2^Department of Biomedicine, University of Bergen, Bergen, Norway; ^3^Department of Molecular and Translational Science, Monash University, Clayton, VIC, Australia; ^4^Cancer and Inflammation Laboratory, Olivia Newton-John Cancer Research Institute, Melbourne, VIC, Australia; ^5^School of Cancer Medicine, La Trobe University, Melbourne, VIC, Australia

**Keywords:** orphan nuclear receptors, breast cancer, transcription factors, breast cancer subtypes, small molecule inhibitors

## Abstract

There is emerging evidence asserting the importance of orphan nuclear receptors (ONRs) in cancer initiation and progression. In breast cancer, there is a lot unknown about ONRs in terms of their expression profile and their transcriptional targets in the various stages of tumor progression. With the classification of breast tumors into distinct molecular subtypes, we assess ONR expression in the different breast cancer subtypes and with patient outcomes. Complementing this, we review evidence implicating ONR-dependent molecular pathways in breast cancer progression to identify candidate ONRs as potential prognostic markers and/or as therapeutic targets.

## Introduction

Breast cancer is the most common cancer in women worldwide. Nearly 1.7 million new cases diagnosed in 2012 accounted for 12% of all new cancer cases and 25% of all cancers in women (1). The incidence of breast cancer is expected to increase primarily due to changes to the demographic with increasing aging populations and obesity, key risk factors for breast cancer. While patients with primary breast cancer are treated successfully, it is in the treatment of recurrent and invasive tumors where there is a clear need for the development of new therapies. Here, we discuss the potential of members of the nuclear receptor (NR) family as druggable targets for the discovery of new treatments in breast cancer.

The growth of the primary tumor in the breast is predominantly dependent on estrogen (2), via its interaction with its target receptor, the estrogen receptor (ER), expressed in 75% of all breast cancers. Hence, endocrine therapy is currently the most effective adjuvant treatment for ER-positive breast cancer. These include selective ER modulators (SERMs), selective ER down-regulators (SERDs), and aromatase inhibitors (AIs) all of which target the estrogen action and biosynthesis pathways (3–5). Despite the success of endocrine treatments in breast cancer, patients often present with resistance to endocrine therapy, despite high tumor ER expression. Furthermore, approximately 70% of patients with advanced disease ultimately acquire resistance to endocrine therapy [reviewed by Musgrove and Sutherland (6)]. The mechanisms for *de novo* or acquired endocrine therapy resistance are still poorly understood and may involve mechanisms of crosstalk between ER and other cell signaling networks, e.g., the Human Epidermal Growth Factor Receptor (HER) pathways. The actions of ER, Progesterone Receptor (PR), and Androgen Receptor (AR) in mediating breast cancer growth are well described in the literature. However, often overlooked in this context are the other members of the NR superfamily that function as transcription factors in regulating steroidogenesis, proliferation, and invasion and migration properties of cancer cells. The focus of the current review is to interrogate the expression patterns of the orphan nuclear receptors (ONR) and “adopted ONRs” in breast cancer, identify correlations with patient outcomes, and review the literature for functional evidence that may identify new ONRs as potentially linked to breast cancer in a prognostic sense or potential therapeutic targets.

Orphan NRs, referred to as ONRs throughout this review (Table 1), form a subgroup within the NR superfamily (7). Unlike the NRs, that are ligand activated; the ONRs have been classified together due to the lack of known endogenous ligands or other interacting synthetic compounds and drugs. Table 1 provides an overview of the ONR and adopted ONRs along with the potential endogenous or synthetic ligands that have been identified. While functioning in a similar manner to NRs, the ONRs can dimerize and bind to NR response elements (consisting of two palindromic NR half sites) to mediate transcription. As with NRs, ONRs can also hetero-dimerize, however it is ONRs that bind to NR half sites as monomers. Within the ONRs, the term “adopted ONRs” describes ONRs for which endogenous ligand or synthetic compounds have now been identified and shown to modulate activity (7–9) (Table 1). Structural analysis of ONRs also demonstrates that certain NRs, such as NR5A1 and NR5A2 are held in a constitutively active confirmation to maintain ligand-independent activity (10–14). Furthermore, modulation of the activity of NRs and ONRs by co-regulator proteins such as Nuclear Receptor Co-activators (NCOAs 1–3, also termed steroid receptor co-activators or SRCs 1–3), form an essential component of functional modulation to dictate whether genes are actively transcribed or repressed (15–17). The identification of synthetic compounds to modulate ONR activity is testament to the druggability of this class of transcription factors making them an attractive target for drug development in cancer. Due to their druggability and wide-ranging cellular functions, there is considerable interest in identifying novel ligands for ONRs as a therapeutic tool.

**Table 1:** **Orphan nuclear receptors and proposed ligands**.

Gene symbol	Common name	Potential ligand(s)	Reference
NR0B1	DAX-1	Not known	
NR0B2	SHP	CD437 Retinoids^a^	(17–20)
NR1D1	Rev-Erbα	Heme^a^	(21–25)
NR1D2	Rev-Erbβ	Heme^a^	(21–25)
NR1F1	RORα	Isoflavones, Cholesterol, derivatives	(26–28)
NR1F2	RORβ	all-trans retinoic acid (ATRA)	(29)
NR1F3	RORγ	Isoflavones	(28)
NR2A1	HNF4α	Fatty acids	(30, 31)
NR2A2	HNF4γ	Fatty acids	(31)
NR2C1	TR2	Not known	
NR2C2	TR4	Retinol/ATRA^a^	(32)
NR2E1	TLX	Ccrps: small molecule agonists	(33)
NR2E3	PNR	Benzimidazoles	(34, 35)
NR2F1	COUP-TF-I	Not known	
NR2F2	COUP-TF-II	Retinol/ATRA^a^	(36)
NR2F6	EAR2	Not known	
NR3B1	ERRα	Isoflavones, Diethylstilbestrol (DES), Chlordane	(37–39)
NR3B2	ERRβ	Isoflavones, DES, 4-Hydroxy tamoxifen (4HT)	(37, 38)
NR3B3	ERR gamma	Isoflavones, DES, 4HT	(37, 38, 40)
NR4A1	NGFI-B	Cytosporone B^a^, Diindolylmethane Analogs (C-DMIs)	(41–47)
NR4A2	NURR1	Benzimidazoles, C-DMIs, 6-Mercaptopurine	(48–51)
NR4A3	NOR1	6-Mercaptopurine, Prostaglandin A2	(52, 53)
NR5A1	SF-1	Phospholipids	(11, 12)
NR5A2	LRH-1	Phospholipids	(11, 54)
NR6A1	GCNF	Not known	

*^a^Compounds or proteins that bind the ONR*.

*Potential ligands are defined as those proposed to bind and modulate ONR transcriptional activity*.

Given the roles of ONRs in regulating transcription of genes involved in processes important for development, metabolism, immunity, angiogenesis, steroidogenesis, and fertility, their actions have also been implicated in multiple diseases including diverse types of cancers. Although not widely reported, there is emerging but clear evidence of ONR-dependent mechanisms in the regulation of tumor growth and progression. Recent work quantifying the expression profiles of the NR superfamily members in well curated tumor biopsy tissue and isolated cancer-associated fibroblast (CAFs) now allow a good insight into the possible actions of NRs in the development and progression of breast cancer (55–57).

We analyzed datasets available online to establish correlations between ONR expression and clinical parameters that define breast cancer outcomes. We also present an overview of the current understanding of ONR-related cellular mechanisms in breast cancer development.

## Molecular Signatures Defining Breast Cancers

There are now several intrinsic molecular signatures acquired by genome-wide expression profiling of breast tumors that are used to stratify the heterogeneity of breast cancers (58–62). A clear delineation of these multi-gene signatures will ultimately allow personalized therapies. Breast tumors are classified into a variety of molecular subtypes with the four major subtypes being Luminal A, Luminal B, HER2-enriched, and basal-like tumors. Luminal A and Luminal B subtypes are both ER-positive tumors; Luminal A tumors being less aggressive than the Luminal B subtype, and more responsive to endocrine therapies (63–66). HER2-enriched tumors show amplification of the *HER2* gene and these patients respond well to treatment that target HER2. High ER and PR levels, the lack of *HER2* amplification in low-grade tumors and specific gene signatures, are now used as indicators of good effectiveness with endocrine therapy (61, 62). Basal-like tumors, also known as Triple-Negative Breast Cancers (TNBCs, lacking expression of ER, PR and HER2) are aggressive tumors with the only treatment options being surgery and chemotherapy.

In this vein, to address the question that other NRs could be used to further classify tumor subtypes, recently, three key studies provide a comprehensive expression profile of the NR superfamily and their interacting co-regulatory proteins in tumor and associated stroma in breast cancer (55–57). Based on these publications, akin to ER status, the expression of other NRs and ONRs could very likely provide additional prognostic power in the classification of breast tumor subtypes.

## Correlation of ONR mRNA Transcript Expression with Patient Survival Outcomes

We used publically available Kaplan–Meier Plotter^1^ and BreastMark^2^ algorithms to correlate ONR gene expression to clinical outcomes within the datasets available within the two platforms as indicated in Tables 2 and 3. Kaplan–Meier Plot analysis was used specifically to assess ONR expression in relation to relapse free survival (RFS) in breast cancer patients; analysis also stratified to ER-positive and ER-negative status from gene expression data and survival information of 1,809 patients collated from GEO (Affymetrix HGU133A and HGU133 Plus 2.0 microarrays) EGA and TCGA databases (67). With the BreastMark algorithm, mRNA expression patterns of the genes of interest can be classified within breast cancer subtypes according to the PAM50 molecular signature (68). The database integrates gene expression and survival data from 26 datasets from 12 microarray platforms corresponding to 4,738 samples. We utilized the database to identify ONRs in which mRNA expression levels were positively or negatively correlated with disease progression in all breast cancer or stratified into the various subtypes.

**Table 2:** **Correlation of ONR expression to DFS in breast cancer patients**.

Gene symbol	Common name	As a whole	Node pos	Node neg	LumA	LumB	Her2 pos	Basal
NR0B1	DAX-1	ns	ns	ns	ns	ns	ns	ns
NR0B2	SHP	ns	ns	*p* = 0.022^#^	ns	*p* = 0.004^#^	ns	ns
NR1D1	REV-ERB alpha	ns	ns	ns	ns	*p* = 0.015*	ns	ns
NR1D2	REV-ERB beta	ns	ns	ns	ns	ns	ns	*p* = 0.030^#^
NR1F1	ROR alpha	ns	ns	ns	ns	ns	ns	ns
NR1F2	ROR beta	ns	ns	ns	ns	*p* = 0.011^#^	ns	ns
NR1F3	ROR gamma	ns	ns	ns	ns	ns	ns	ns
NR2A1	HNF4 alpha	ns	ns	ns	*p* = 0.035*	ns	ns	ns
NR2A2	HNF4 gamma	*p* = 0.001*	ns	ns	ns	ns	ns	*p* = 0.023*
NR2C1	TR2	ns	ns	ns	ns	*p* = 0.035*	ns	ns
NR2C2	TR4	ns	ns	ns	ns	ns	ns	ns
NR2E1	TLX	ns	ns	ns	ns	*p* = 0.003^#^	ns	ns
NR2E3**	PNR							
NR2F1	COUP-TF-I	ns	ns	ns	ns	ns	ns	ns
NR2F2	COUP-TF-II	ns	*p* = 0.009^#^	ns	ns	ns	ns	ns
NR2F6	EAR2	ns	ns	ns	*p* = 0.038^#^	ns	ns	ns
NR3B1	ERR alpha	*p* = 0.009*	*p* = 0.019*	ns	ns	ns	ns	ns
NR3B2	ERR beta	ns	ns	*p* = 0.01^#^	ns	ns	ns	ns
NR3B3	ERR gamma	ns	*p* = 0.01^#^	ns	ns	ns	ns	ns
NR4A1	NUR77	ns	ns	ns	ns	ns	ns	ns
NR4A2	NURR1	*p* = 0.003^#^	*p* = 0.007^#^	ns	ns	ns	ns	ns
NR4A3	NOR1	ns	*p* = 0.003^#^	ns	ns	ns	ns	ns
NR5A1	SF-1	ns	ns	ns	ns	*p* = 0.025^#^	ns	ns
NR5A2	LRH-1	ns	ns	ns	ns	*p* = 0.003*	ns	ns
NR6A1	GCNF	ns	ns	*p* = 0.015^#^	ns	ns	ns	ns

*Based on BreastMark mRNA analysis (http://glados.ucd.ie/BreastMark/mRNA_analysis.html)*.

*Significance; *p* < 0.05, ns = non-significant; *p* ≥ 0.05*.

*^#^Higher ONR expression is significantly correlated with increased survival in BCa patients*.

*Lower ONR expression is significantly correlated with increased survival in BCa patients.

**Not included in the database. DFS, disease free survival; BCa, breast cancer.

**Table 3:** **Correlation of ONR expression to RFS in breast cancer patients**.

Gene symbol	Common name	All BCa	ER-positive BCa	ER-negative BCa	Affy ID
NR0B1	DAX-1	ns	ns	*p* = 0.0001^#^	206645_s_at
NR0B2	SHP	*p* = 3.5e^−7#^	ns	ns	206410_at
NR1D1	REV-ERB alpha	*p* = 9.1e^−5#^	ns	ns	31637_s_at
NR1D2	REV-ERB beta	*p* = 1e^−4^*	ns	ns	209750_at
NR1F1	ROR alpha	*p* = 0.004^#^	ns	ns	210426_x_at
NR1F2	ROR beta	*p* = 0.0002^#^	ns	ns	206443_at
NR1F3	ROR gamma	*p* = 0^#^	*p* = 0.002^#^	ns	206419_at
NR2A1	HNF4 alpha	*p* = 2.9^−5#^	ns	ns	208429_x_at
NR2A2	HNF4 gamma	*p* = 1.8e^−6#^	ns	ns	207456_at
NR2C1	TR2	ns	ns	ns	204791_at
NR2C2	TR4	*p* = 0.0036^#^	ns	ns	206038_s_at
NR2E1	TLX	*p* = 0.0062^#^	ns	*p* = 0.0008^#^	207443_at
NR2E3	PNR	ns	*p* = 0.0036^#^	ns	208385_at
NR2F1	COUP-TF-I	*p* = 0.0029^#^	ns	ns	209506_s_at
NR2F2	COUP-TF-II	ns	ns	*p* = 0.034*	209120_at
NR2F6	EAR2	ns	ns	ns	209262_s_at
NR3B1	ERR alpha	ns	ns	ns	1487_at
NR3B2	ERR beta	*p* = 2.2e^−7#^	ns	ns	207726_at
NR3B3**	ERR gamma				Not available
NR4A1	NUR77	*p* = 1.5e^−5#^	*p* = 0.013^#^	ns	202340_x_at
NR4A2	NURR1	*p* = 1.7e^−8#^	*p* = 0.0054^#^	ns	216248_s_at
NR4A3	NOR1	*P* = 0.0067^#^	*p* = 0.014^#^	ns	209959_at
NR5A1	SF-1	*p* = 0.046^#^	ns	ns	210333_at
NR5A2	LRH-1	*p* = 5.8e^−12#^	ns	ns	208337_s_at
NR6A1	GCNF	*p* = 0.0003^#^	ns	ns	207742_s_at

*Table is based on KM Plot per April 2015 (http://kmplot.com/analysis/index.php?p=service&cancer=breast)*.

Significance; *p* < 0.05, ns = non-significant; *p* ≥ 0.05

*^#^Higher ONR expression is significantly correlated with increased survival in BCa patients*.

*Lower ONR expression is significantly correlated with increased survival in BCa patients.

**Not included in the database. RFS, relapse free survival; BCa, breast cancer.

### ONRs associated with the luminal A subtype

Lower expression of Hepatocyte Nuclear Factor 4 alpha (HNFα, NR2A1) and higher expression of V-erbA related protein (EAR2, NR2F6) were associated with increased DFS in patients with the Luminal A subtype of breast cancer (*p* = 0.035 and *p* = 0.038, respectively, Table 2). The functions of these two ONRs in the context of breast cancer are not defined as yet, although HNF4α, a key transcriptional regulator of hepatocyte differentiation and function in liver, acts as a tumor suppressor in the diethylinitrosamine-induced rat model of liver carcinogenesis, and inhibits epithelial-mesenchymal transition (EMT) (69). In a recent study focusing on expression patterns of genes associated with altered metabolism of breast cancer cells (using the TCGA database), the analysis of 556 transcription factor sequence motifs identified HNF4α-specific response elements as enriched in differentially co-expressed gene targets (70). Interestingly, this data suggest a potential function for this ONR in breast cancer cells in mediating tumor proliferation via the regulation of cellular metabolism, and warrants further investigation.

EAR2 is a member of the COUP-TF orphan subfamily and its established functions include negative regulation of renin and the luteinizing hormone receptor gene transcription and brain function including memory and learning (71–74). EAR2 expression is highly expressed in both ER-positive and ER-negative tumors relative to normal breast tissue (57). Additionally, EAR2 has been shown to directly interact with the aromatase promoter and down-regulate the expression of aromatase in breast cancer cell lines (75).

### ONRs associated with the luminal B subtype

Within the BreastMark criteria, reduced expression of REV-ERBα, TR2, LRH-1, and increased RORβ and SHP expression are significantly associated with greater DFS in patients in Luminal B tumors (*p* = 0.015, *p* = 0.035, *p* = 0.003, *p* = 0.011, *p* = 0.004, *p* = 0.025 and *p* = 0.003, respectively, Table 2). In contrast to Luminal A tumors, a greater number ONRs are associated with the Luminal B subtype, a more aggressive tumor and patients are often resistant to treatment. The functional implications of these expression profiles for each ONR associations are discussed in detail below.

REV-ERBα (also known as NR1D1) is a transcriptional repressor, abundantly expressed in adipose tissue and its functions are linked mainly to adipocyte differentiation, regulation of cell metabolism, and thermogenic responses (76) REV-ERBα lacks a transcriptional activation domain and represses target genes via its interaction with the transcriptional corepressor N-CoR. REV-ERBα has been shown to have a prosurvival function in HER2-positive breast cancer cells (77). However, recently it has been shown that REV-ERBβ (NR1D2) is also expressed in breast cancer cell lines and that its expression not correlated with HER2 or ER expression (9). Furthermore, the identification of a novel REV-ERBβ inhibitor, ARN5187, results in tumor cell cytotoxicity suggesting anticancer effects of REV-ERBβ suppression (9). We observed higher expression of REV-ERBβ as significantly associated with increased DFS in the basal cancer subtype (*p* = 0.03). Further determination of the protein expression and function of the two REV-ERB isoforms would allow a better understanding of the roles these ONRs play in the different breast cancer subtypes particularly with regards to HER2 status. Given its significant interaction with multiple co-regulators in ER-negative breast tumors, and a significant correlation to predict poorer outcome for patients, further investigation of the two REV-ERB isoforms in breast cancer is warranted.

Similarly to REV-ERB isoforms, TR2 (NR2C1) is a transcriptional repressor, and inhibits ERα-mediated transcription to regulate cell proliferation in breast cancer cells (78). In transgenic mouse models where TR2 is either over-expressed or down regulated, TR2 was identified as a key transcriptional repressor of GATA1 (79). Therefore, reduced TR2 in tumors may be the mechanism via which increased GATA1 promotes a more aggressive tumor phenotype in breast cancers (80). However, it is the lower expression of TR2 that was associated with increased survival in women with Luminal B tumors (Table 2, *p* = 0.035). Further investigation of the protein localization and correlation to TR2 mRNA expression profiles and the identification of breast cancer specific gene targets are required to further understand this observation.

In contrast to the REV-ERBs and TR2, Liver Receptor Homolog-1 (LRH-1, NR5A2) is a constitutive transcriptional activator, binding DNA as a monomer to promote transcription (11, 12, 14). The activity of LRH-1 was previously thought to be mainly under the control of co-regulators (14, 54, 81, 82, 83); however potential endogenous ligands identified include phosphatidylinositols, in particular, phosphatidylinositol-3-phosphate (PIP_3_) (11, 54), small molecule agonists (84) and antagonists (85).

Low LRH-1 expression significantly correlated with increased DFS in Luminal B tumor patients (Table 2, *p* = 0.003). Additionally, a strong correlation with co-regulator expression in ER-negative breast tumors suggests that increased LRH-1 activity may correlate with predicted poorer outcome for those patients (55). The expression of LRH-1 in both ER-positive and ER-negative tumors indicate that more functional evidence is require to determine its contribution in these varying tumor contexts. There is strong functional evidence linking LRH-1 to estrogen-regulated pathways in breast cancer tumor and associated stromal cell types [reviewed in Ref. (86)].

LRH-1 stimulates transcription of the aromatase gene, thereby promoting estrogen synthesis within CAFs (87–89). Given this strong association with tumor-associated estrogen production, most functional studies have been directed toward understanding the interplay between LRH-1 and estrogen-dependent proliferation pathways within breast tumors. While LRH-1 expression itself is under estrogen regulation, LRH-1 in a positive feedback manner induces estrogen-mediated cell proliferation (90). This is primarily via the co-operative regulation of gene-transcription of ERα and LRH-1 target genes (91–93). Both ERα and LRH-1 directly stimulate the transcription of one another, adding to the close functional relationship between the two NRs (90, 94). There are five transcript variants of LRH-1 that give rise to protein isoforms of three different sizes. There is some evidence that transcript variant four may be the active LRH-1 isoform in ER-positive breast cancer cells (94).

LRH-1 expression is detectable in both ER-positive and negative tumors (95). Despite low mRNA, LRH-1 protein levels are readily detected in ER-negative breast cancer cell lines and tumor tissue (56, 95–97) and this discrepancy is partly explained by increased transcript stability in ER-negative tumor cells (97). Knockdown of LRH-1 expression with shRNA constructs in ER-negative cells reduced cell migration and invasion in ER-negative breast cancer cells (98). The functional importance of LRH-1 in ER-negative cells and the ability of LRH-1 to regulate cell proliferation gene expression in endocrine-resistant breast cancer cells (91) as well as in the presence of SERDs (92) suggests a possible role in promoting tumor-aggressiveness. Given the breadth of knowledge on the tumor promoting effects of LRH-1 in various cancers, LRH-1 is a strong candidate as a therapeutic target. However, there is a need to demonstrate its contribution to mammary tumor initiation and formation using *in vivo* mouse models.

As age is the most common risk factor in breast cancer, it is interesting to note that the expression of RAR-related orphan receptor β (RORβ, also known as NR1F2) is greater in the postmenopausal breast compared to premenopausal tissue. However, its expression is significantly downregulated in ER-negative breast cancer implicating a potential role in the early stages of tumor growth (57). Above median expression of RORβ is correlated with greater DFS rates in patients with Luminal B cancers (Table 2, *p* = 0.011). Although its function is unclear, the ligands for RORβ may provide additional functional evidence of its roles in breast cancer cells.

### ONRs associated with lymph node-positive breast cancer

Lymph node status is one of the most important predictors of breast cancer recurrence and survival. Data from the Surveillance, Epidemiology, and End Results (SEER) Program shows that women with lymph node negative tumors have a better prognosis compared to those with tumors in the lymph nodes.

In addition to overall DFS for all breast cancer cases, low ERRα and high NURR1 expression was associated with improved survival in patients with lymph node positive tumors (*p* = 0.019 and *p* = 0.007, respectively, Table 2). Structurally and functionally related to the ERs, ERRα (Estrogen-related receptor (ERR) alpha, also known as NR3B1) over-expression is associated with a poorer outcome in patients with ER-negative breast cancer (99). Functional *in vitro* studies in breast cancer cell lines show that the suppression of ERRα inhibits cellular proliferation and migration (100). Expression of ERRα positively correlates with HER2 status in breast tumors and ERRα transcriptional activity is positively modulated by EGFR/HER2 signaling in breast cancer cells. This suggests a role for ERRα in mediating the transition from ER-positive luminal toward more aggressive HER2-expressing tumor subtype (101, 102).

NURR1 (the NR-related 1 protein, also known as NR4A2), nerve growth factor I B (NGFIB, also known as Nur77 or NR4A1), and the neuron-derived orphan receptor 1 (NOR1, NR4A3) together constitute the NR4A subfamily of NRs (103–105). No endogenous ligands have been identified for these ONRs and their ability to transactivate genes appears to be constitutive. The NR4A receptors are however targeted by several hormones and xenobiotic compounds that affect the expression and/or activity of these stress early response genes (Table 1) (106). A recent study profiling all NRs in ER-positive and ER-negative breast tumors revealed that while most NR mRNA levels were significantly lower in breast cancer versus normal tissue, the expression of all of the NR4A family members (Nur77, NURR1, and NOR1) were significantly up-regulated in ER-positive breast cancer (57). Additionally in ER-negative breast tumors, when compared to normal breast tissue, NUR77 and NOR-1, together with EAR2, were found to have significantly increased (57).

In primary tumors, protein levels of NURR1 are inversely correlated with lymph node metastasis (107). This observation is mirrored in our analysis with the BreastMark dataset showing increased NURR1 mRNA expression is significantly correlated with increased survival in lymph node positive breast cancer. Data analysis from BreastMark and Kaplan–Meier Plotter indicate above median expression of NURR1 mRNA is associated with better prognosis and RFS in all breast cancer patients (107). However, functional *in vivo* experiments indicate the contrary. When NURR1 expression is knocked down by shRNA, growth of xenografts of highly invasive MDA-MB-231 and MDA-MB-468 breast cancer cells in athymic nude mice is significantly attenuated (107). Interestingly, high cytoplasmic expression of NURR1 is significantly correlated with advanced pathologic stage and higher tumor grade of bladder tumors as well as an increased number of distant metastases and decreased recurrence free survival (108). Thus, not only the expression level of NURR1 but the sub-cellular localization in which it is expressed may be of importance in its contribution toward tumor growth and metastatic potential.

Increased expression of NOR1 is correlated with better clinical outcomes in patients with lymph node positive tumors and ER-positive tumors (*p* = 0.003 and *p* = 0.014 Tables 2 and 3, respectively). Little is known about the actions of NOR1 in breast cancer, however in the ER-positive breast cancer cell line MCF7, NOR1 mRNA is up-regulated by a pro-apoptotic compound A23187 (109), shown to cause apoptotic cell death, suggesting a possible role in tumor cell apoptosis. NOR1 and NUR77 are thought to form heterodimers with NR retinoid X receptor RXR to modulate retinoic acid (RA) signaling (110, 111). Additionally in a positive feedback loop, RAs are involved in the positive regulation of NOR1 and NURR1 and the downregulation of NUR77 in breast cancer cells (112) correlating to decrease cell proliferation (113–115). Taken together these studies indicate that the NR4A family members may exert multiple functions in cancer, through their genomic functions in promoting tumor growth and survival, and, paradoxically, through non-genomic functions that may potentially exert tumor suppression by induction of apoptosis.

We observed an association of increased COUP-TFII expression with improved survival in patients with lymph node-positive tumors (Table 2, *p* = 0.009). The mRNA levels of the COUP transcription factors COUP-TFI (NR2F1) and COUP-TFII (NR2F2) were reported to be significantly decreased in both ER-positive and ER-negative breast cancer tissue compared to normal breast (57). COUP-TFII mRNA showed a discriminating role in the classification of breast cancer grades as it was more highly expressed in better-differentiated, lower-grade lesions (57). COUP-TFII was, in addition to the NR members TRβ, MR and PPARγ, reported to be a predictor of improved metastasis-free survival in tamoxifen-treated patients after adjustment for expression of ERα (57).

However, previously the amounts of protein of both these receptors have been reported to be higher in human breast cancer tissue than normal breast (116, 117). While the results are uncertain for COUP-TFI as it was only examined in a very small set of samples (116), COUP-TFII protein expression has been examined in a larger cohort of human breast cancers (117). Of 119 invasive ductal carcinoma of the breast, 59% stained positive for COUP-TFII in the nuclei of carcinoma cells (117). Higher nuclear expression of COUP-TFII was correlated with decreased disease-free and overall survival of the patient (117). Additionally, COUP-TFII was correlated to clinical stage (significantly higher expressed in stage IV tumors than stage I), histological grade (significantly higher expression with grade) and ERα status (significantly higher expressed in ERα positive than negative breast carcinomas) (117). Several groups have looked at the function of COUP-TFI and COUP-TFII in breast cancer cell lines. COUP-TFI may promote estrogen-independent transcriptional activity of ERα in breast cancer cells (116, 118). Furthermore, COUP-TFI mediates its actions through CXCL12/CXCR4 signaling. The over-expression of COUP-TFI results in down-regulation of CXCL12 and up-regulation of CXCR4 expression, to promote motility of MCF-7 cells (119). Furthermore, COUP-TFI mRNA expression is significantly higher in grade 1 (but not in grade 2 or 3) tumors than in normal breast tissue and that CXCR4 mRNA is significantly higher in cancerous tissue than in normal breast tissue and its expression is increasing with tumor grade, whereas transcripts of CXCL12 was significantly decreased in all the tumor grades compared to normal tissues (119).

### ONRs associated with lymph node-negative breast cancer

Higher SHP, ERRβ and GCNF expression were associated with improved survival in patients with lymph node-negative tumors (*p* = 0.022, *p* = 0.01, *p* = 0.015, respectively, Table 2). Like Dax-1 (NROB1), the other member of this subgroup, short heterodimer partner (SHP, also termed NROB2) lacks a DNA-binding domain (DBD) and represses the action of NRs, including ERα through direct interaction with the receptor (120). SHP has been shown to inhibit aromatase expression by repressing LRH-1 activity on the promoter II element of *Cyp19*, the gene encoding aromatase (121). The actions of SHP in the repression of ER, LRH-1 and other NRs illustrates the importance of assessing the expression profile of this ONR in relation to other NRs to gage NR functionality. Above median expression of SHP also correlated with increased DFS in the luminal B subtype (Table 2, *p* = 0.004). These findings indicate that SHP may have a protective function in breast cancers and may be specifically so in lymph node-negative and luminal B subtype tumors. The expression and further characterization of this ONR are important due to its interactions with a range of NRs; whether SHP binds preferentially to ONRs over other co-regulators would also assist in defining its contribution to ONR-dependent actions in the cancer context.

Estrogen-related receptors are a subgroup of ONRs that have a strong homology with ER. We have previously discussed the roles of ERRα in lymph node positive tumors. Above median expression of ERRα is associated with greater survival in patients with lymph node-negative tumors. In other studies, the overexpression of ERRβ has been correlated with improved prognosis and longer relapse-free survival in breast cancer patients, and its mRNA levels inversely correlated with tumor cells in S-phase in patient-derived tumor samples (122). The functions of ERRs are tightly related to ER-mediated actions in breast cancer cells mainly due to the high degree of structural similarity to one another and to ER (123, 124), there is very little known about the details of ERR family members in this context. Recently though, in a detailed comparison of the ERRs on estradiol-stimulated ER transcription it was identified that it is only ERRβ that co-localized into the nucleus with ERα and with fluorescence resonance energy transfer revealed that ERRβ directly interacted with ERα to repress its transcriptional activity (125). The findings further suggest a unique inhibitory role for ERRβ in estrogen-dependent cellular function such as cancer cell proliferation via the regulation of cell cycle progression.

Similarly above median expression of Germ Cell Nuclear Factor Receptor (GCNF) is correlated with improved patient outcomes compared to those with below median expression (Table 2, *p* = 0.015). Initially identified as exclusively expressed in germ cells (126), GCNF is well characterized for its importance in embryonic development in regulating neural development and gastrulation (127) and oocyte function (128). Its unlikely role in breast cancer is suggested from a recent analysis of Affymetrix, Illumina and RNAseq microarray data to perform gene enrichment analysis identified shared common promoter motifs for GNCF in TNBC samples (129).

## ONRs in ER-Positive and ER-Negative Breast Cancer

The Kaplan–Meier Plot analysis was used to assess ONR expression in relation to RFS in breast cancer patients; stratified to ER-positive and ER-negative tumors. Here, gene expression data and survival information of 1,809 patients have been collated from GEO (Affymetrix HGU133A and HGU133 + 2 microarrays) EGA and TCGA (130). Above median expression of 16 out of 24 ONRs was significantly correlated with increased RFS when analyzing all breast cancer patients in the database (Table 3, *n* = 3554). As an exception, lower expression of REV-ERBβ was correlated with increased patient RFS (*p* = 1.0e^−4^). In ER-positive breast cancer, above median expression of five ONRs including RORγ, PNR, Nurr77, Nurr1 and NOR1 was associated with better RFS (*p* = 0.002, *p* = 0.0036, *p* = 0.013, *p* = 0.0054 and *p* = 0.014, respectively, Table 3). In ER-negative breast cancer patients, higher expression of Dax-1 and TLX, and lower COUP-TFII expression were found to significantly correlate with increased RFS of (*p* = 0.0001, *p* = 0.0008, and *p* = 0.034, respectively, Table 3). This pattern or ­association with ER status may be important in identifying the ­regulatory mechanisms via which these ONRs are expressed; whether these ONRs modify/enhance the responses to endocrine therapies directed at ER such as SERMs are yet to be delineated.

## Summary

The NR family of transcription factors plays diverse and important roles in development and the regulation of normal physiological functions in a tissue-specific manner. By understanding the contribution of ONRs in breast cancer subtypes, many novel mechanistic insights into tumor progression can be gleaned. From our data analysis, we observe complex expression patterns of various ONRs that could be selectively assessed in Luminal A, Luminal B, Lymph node positive and negative tumor types. Indeed within the specific subtype, the function of a particular ONR may possess a completely unique transcriptional imprint, therefore with varied effects on tumor proliferation and invasiveness; as is observed for AR, which can have pro- and anti-proliferative effects that is dependent on ER status of the breast cancer cell.

There is a lot that is unknown about ONRs in terms of their basic expression profile, mode of regulation of expression, cell subtype expression patterns, roles in normal mammary gland development processes, and transcriptional targets in the mammary gland and tumor scenarios. Our review attempted to identify “candidates” that were closely associated with breast cancer survival outcomes and whether molecular subtypes of breast cancer could be linked to ONR expression patterns. In addition to expression patterns, large-scale ChIP-seq experiments in well defined in breast cancer tissue samples and in *in vivo* tumor models will identify ONRs that are critical in this process. In addition, determining changes in ONR expression linked to key risk factors in breast cancer may also assist in understanding the mechanisms of this disease. The ligand-dependent actions of NRs provide extensive information that could potentially allow us to design new ligands for the ONRs in order to modulate their function. By the pharmacological targeting critical ONRs, we may in future identify effective therapies that could complement existing therapies in the treatment of breast cancer.

## Conflict of Interest Statement

The authors declare that the research was conducted in the absence of any commercial or financial relationships that could be construed as a potential conflict of interest.

## Footnotes

^1^www.kmplot.com

^2^http://glados.ucd.ie/BreastMark/index.html
